# Impact of kidney injury on fluid overload and impaired oxygenation

**DOI:** 10.1186/cc14374

**Published:** 2015-03-16

**Authors:** A Akcan Arikan, LL Loftis, MA Arnold, CE Kennedy

**Affiliations:** 1 Baylor College of Medicine, Houston, TX, USA

## Introduction

Severity of acute kidney injury (AKI) and fluid overload (FO) are not incorporated into current severity of illness measures and are invisible to the practitioner. The causal relationship and timing between AKI and FO and oxygenation is not clear. The Fluid Overload Kidney Injury Score (FOKIS) is a daily score incorporating subscores for AKI (pRIFLE (creatinine (Cr) and urine output (UOP))), FO (total fluid (in - out) / ICU admission weight) >15% in five percentile increments, and exposure to nephrotoxic medications. We previously reported that FOKIS outperforms PRISM in mortality prediction in our pediatric intensive care unit (PICU). We hypothesized that patients with AKI on admission to the PICU developed worse fluid overload and in turn worse oxygenation.

## Methods

We prospectively calculated daily FOKIS scores and subscores (Cr, UOP, FO) in PICU patients. We excluded patients with <7 day stays in order to properly explore the association between timing of AKI and FO and oxygenation by oxygenation index (OI).

## Results

Over 18 months, there were 2,830 patients, 436 patients with PICU stay >7 days, 361 patients had complete data for all 7 days. Mortality was 4.5% overall and 11% cohort. A total of 246 patients (68%) had AKI (by FOKISCr or FOKISuop); 205 patients (57%) on day 1, 85 patients (24%) on day 3. Admission or day 3 AKI by either FOKIS subscore (FOKISCr or FOKISuop)) did not predict maxFO or mortality. Increasing total FOKIS score was associated with increasing mortality and increasing OI (Table 1). FOKIS, controlled for PRISM, was an independent predictor of OI (*P *= 0.03).

## Conclusion

In PICU patients, admission or day 3 AKI alone did not predict maxFO. A composite score that includes both AKI and FO parameters correlated with OI and discriminated survivors from nonsurvivors. FO seems to result from combination of increased fluid exposure with underlying AKI but cannot fully be explained by oliguria in pediatric patients.

